# Volvulus grêlique sur hydatidose péritonéale: une cause rare d'occlusion

**DOI:** 10.11604/pamj.2014.18.79.4205

**Published:** 2014-05-24

**Authors:** Abdesslam Bouassria, Khalid Mazine, Hicham Elbouhaddouti, Ouadii Mouaqit, Abdelmalek Ousadden, Khalid Mazaz, Elbachir Benjelloun, Khalid Ait Taleb

**Affiliations:** 1 Service de Chirurgie Viscérale, CHU Hassan II – Fès, Université Sidi Mohamed Ben Abdellah, Faculté de Médecine et de Pharmacie de Fès, Maroc

**Keywords:** Hydatidose péritonéale, volvulus grêlique, chirurgie, albendazole, peritoneal hydatid disease, small bowel volvulus, surgery, albendazole

## Abstract

L'hydatidose péritonéale peut être primitive, hématogène ou hétérotopique, comme elle peut être secondaire et résulte de la fissuration d'un kyste hydatique, le plus souvent hépatique. Cliniquement polymorphe, elle peut se révéler par des douleurs abdominales ou par la palpation d'une masse abdominale. Nous rapportons le cas d'une patiente chez qui l'hydatidose péritonéale était révélée par un accident occlusif: un volvulus grêlique dû à un volumineux kyste hydatique mésentérique. Le traitement de l'hydatidose péritonéale est chirurgical, couplé à un traitement médical à base d'albendazole.

## Introduction

L'hydatidose ou maladie hydatique s'intègre au sein des cestodoses larvaires. C'est une zoonose complexe qui affecte accidentellement l'homme. Aucune parasitose ne peut toucher l'organisme avec un aussi large éventail de localisations, même si la maladie prédomine au foie et au poumon. L'hydatidose est de plus en plus souvent de découverte fortuite. Le diagnostic repose sur la clinique, mais surtout sur l'imagerie, en premier lieu l’échographie. Le traitement reste chirurgical, même si l'avènement de médicaments imidazolés et du traitement percutané modifie la prise en charge des patients.

## Patient et observation

**Figure 1:**
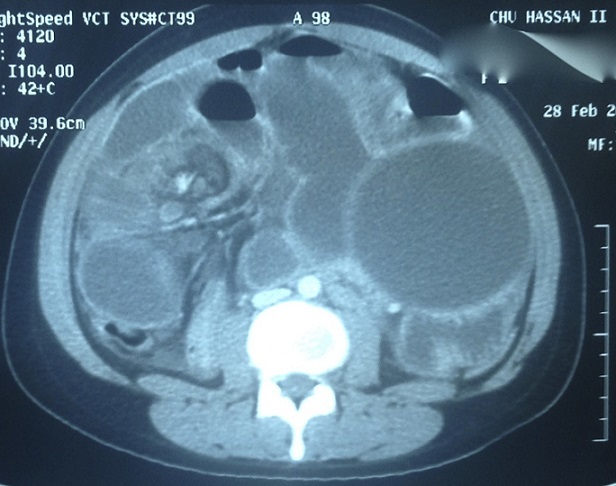
TDM abdominale montrant le volvulus grêlique et le kyste hydatique peritoneal

**Figure 2:**
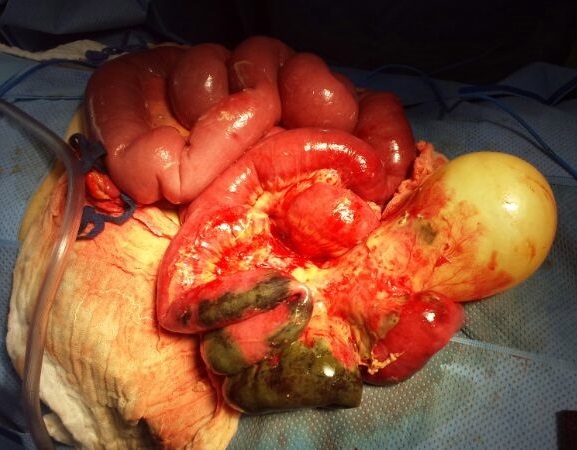
Vue per opératoire du volvulus grêlique et du volumineux kyste hydatique développé aux dépens du mésentèr

**Figure 3:**
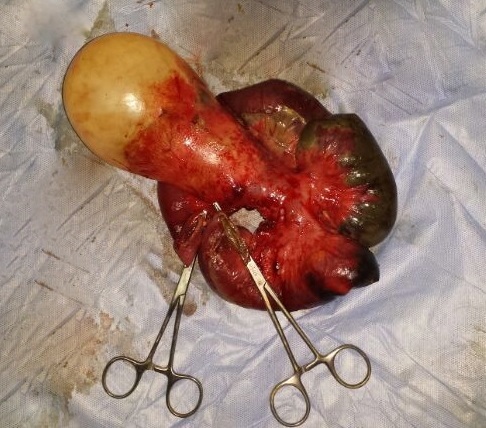
Pièce de résection grêlique emportant le volumineux kyste hydatique mésentérique

Une patiente âgée de 24 ans, opérée il y a 4 ans pour kyste hydatique pulmonaire et cérébral, était admise au service des urgences pour occlusion haute évoluant depuis 3 jours. A l'examen clinique, l'abdomen était distendu et tympanique, et l'ampoule rectale était vide au toucher rectal. Le bilan biologique objectivait une hyperleucocytose à 13900 GB/mm3, et une CRP élevée a 65. Au scanner abdominal, il s'agissait d'une occlusion en rapport avec un volvulus grêlique de la dernière anse iléale. La zone de striction venait au contact d'un volumineux kyste hydatique localisé en fosse iliaque gauche (Figure 1). Le scanner objectivait par ailleurs 3 kystes hydatiques intra péritonéaux dont un pelvien. La patiente était admise en urgence au bloc opératoire. L'exploration chirurgicale retrouvait un volvulus grêlique réalisant 3 tours de spire autour d'un volumineux kyste hydatique développé aux dépends du mésentère des dernières anses iléales (Figure 2). L'anse volvulée était nécrosée sur environ 40 cm, sa paroi était sphacélée par endroits, et il y avait un épanchement intra péritonéal purulent de faible abondance. Par ailleurs, il existait deux autres petits kystes hydatiques mésentériques, et un kyste hydatique pelvien adhérent à la trompe droite. Une résection intestinale emportant le grêle nécrosé et le volumineux kyste hydatique responsable du volvulus était réalisée (Figure 3), suivie d'une double stomie iléale type Bouilly Volkman. Les 3 autres kystes hydatiques intra péritonéaux étaient réséqués. Les suites post opératoires étaient simples, et la patiente, mise sous albendazole était déclarée sortante à J + 5. Notre patiente à été revue en consultation à un mois, elle n'avait pas de plaintes fonctionnelles et l'examen clinique était normal. Un rétablissement de la continuité digestive est programmé à 3 mois.

## Discussion

La fréquence de l'hydatidose péritonéale varie entre 4 et 7% [1, 2]. L'hydatidose péritonéale peut être primitive ou secondaire: la forme primitive est exceptionnelle et se fait soit par voie hématogène, soit par voie hétérotopique par migration en bloc d'un kyste hydatique le plus souvent hépatique, ayant rompu son adventice et gardé sa membrane intacte [3, 4]. La forme secondaire est souvent due à une fissuration ou une rupture d'un kyste hydatique hépatique, plus exceptionnellement splénique [5]. Chez notre patiente, il n'y avait pas de kyste hydatique hépatique, il s'agissait d'une hydatidose primitive. L’échinococcose péritonéale est cliniquement polymorphe et ceci s'explique par l'extrême diversité des localisations des kystes hydatiques. La douleur abdominale peut être diffuse, violente et lancinante [6], comme elle peut être atténuée et persistante pendant plusieurs semaines. La symptomatologie tumorale abdominale peut être retrouvée (palpation d'une masse abdomino-pelvienne rénitente mobilisable et indolore) et doit faire évoquer le diagnostic d'hydatidose péritonéale chez un sujet en bon état général, surtout si elle est associée à une masse hépatique [4]. Les phénomènes compressifs, à type de sub-occlusion [7], d'hypertension portale, d'ictère rétentionnel [8] ou de compression urinaire peuvent être une circonstance de découverte. Les examens biologiques ne sont pas spécifiques. L'hyper éosinophilie peut être remarquée surtout lors d'une fissure ou d'une rupture. L'imagerie médicale est une étape essentielle dans le diagnostic de l'hydatidose. La radiographie standard peut mettre en évidence des déformations des coupoles ou des calcifications limitées en cas de kystes vieillis. L’échographie joue un rôle primordial dans le diagnostic des localisations hydatiques abdominales [8]. Sa fiabilité diagnostique varie de 90 à 100% [1]. La tomodensitométrie a révolutionné l'approche diagnostique lésionnelle et topographique en matière d'hydatidose abdominale. Sa fiabilité topographique excellente, elle permet une meilleure détection des complications et une bonne étude des rapports avec les vaisseaux et l'arbre urinaire. C'est le scanner abdominal qui a permis d'obtenir le diagnostic étiologique et topographique précis chez notre patiente. Le traitement est essentiellement chirurgical [5] et a pour but de traiter en même temps les kystes péritonéaux et le kyste hydatique primitif. La voie d'abord doit réaliser une exposition correcte des kystes, permettre une exploration abdominale aisée et traiter les lésions associées. La laparotomie médiane est la plus utilisée [5]. En matière de kystes hydatiques péritonéaux, c'est le grand nombre des greffes péritonéales qui fait la difficulté de l'intervention [9]. En matière de traitement médical, il faut savoir que la contamination du péritoine représente l'une de ses meilleures indications. Il pourrait prévenir l'apparition d'une échinococcose péritonéale secondaire, difficile à guérir [10]. Différents auteurs ont préconisé l'albendazole dans les cas inopérables et en complément de la chirurgie.

## Conclusion

L’échinococcose péritonéale est souvent secondaire à une rupture ou à une fissure discrète d'un kyste hydatique hépatique. Le tableau clinique est très polymorphe, et l'imagerie médicale est une étape essentielle dans le diagnostic de l'hydatidose, fondée sur l’échographie et la TDM. Le traitement est chirurgical, couplé dans quelques cas à un traitement médical à base d'albendazole. Le pronostic de l'hydatidose péritonéale diffuse reste assez sombre et c'est la précocité du diagnostic et du traitement des localisations primitives qui peut diminuer cette complication.
